# *Notes from the Field*: Mucormycosis Cases During the COVID**-**19 Pandemic — Honduras, May–September 2021

**DOI:** 10.15585/mmwr.mm7050a2

**Published:** 2021-12-17

**Authors:** Homer Mejía-Santos, Sandra Montoya, Rafael Chacón-Fuentes, Emily Zielinski-Gutierrez, Beatriz Lopez, Mariangeli F. Ning, Nasim Farach, Fany García-Coto, David S. Rodríguez-Araujo, Karla Rosales-Pavón, Gustavo Urbina, Ana Carolina Rivera, Rodolfo Peña, Amy Tovar, Mitzi Castro Paz, Roque Lopez, Fabian Pardo-Cruz, Carol Mendez, Angel Flores, Mirna Varela, Tom Chiller, Brendan R. Jackson, Alexander Jordan, Meghan Lyman, Mitsuru Toda, Diego H. Caceres, Jeremy A. W. Gold

**Affiliations:** ^1^Health Surveillance Unit, Secretary of Health of Honduras, Tegucigalpa, Honduras; ^2^Department of Mycology, Hospital Escuela, Tegucigalpa, Honduras; ^3^Central America Regional Office, CDC; ^4^Council of Ministers of Health of Central America and the Dominican Republic, Tegucigalpa, Honduras; ^5^Honduras Country Office, Pan American Health Organization, Tegucigalpa, Honduras; ^6^National Public Health Laboratory, Secretary of Health of Honduras, Tegucigalpa, Honduras; ^7^Honduras Field Epidemiology Training Program, Tegucigalpa, Honduras; ^8^Mycotic Diseases Branch, CDC; ^9^Department of Medical Microbiology, Radboud University Medical Center and Center of Expertise in Mycology Radboudumc/Canisius Wilhelmina Ziekenhuis, Nijmegen, The Netherlands.

On July 15, 2021, the Secretary of Health of Honduras (SHH) was notified of an unexpected number of mucormycosis cases among COVID-19 patients. SHH partnered with the Honduras Field Epidemiology Training Program, the Executive Secretariat of the Council of Ministers of Health of Central America and the Dominican Republic (SE-COMISCA), Pan American Health Organization (PAHO), and CDC to investigate mucormycosis cases at four geographically distinct hospitals in Honduras.

Mucormycosis is a severe, often fatal disease caused by infection with angioinvasive molds belonging to the order Mucorales. Risk factors for mucormycosis include certain underlying medical conditions (e.g., hematologic malignancy, stem cell or solid organ transplantation, or uncontrolled diabetes) and the use of certain immunosuppressive medications (*1*). COVID-19 might increase mucormycosis risk because of COVID-19–induced immune dysregulation or associated medical treatments, such as systemic corticosteroids and other immunomodulatory drugs (e.g., tocilizumab), which impair the immune response against mold infections (*2*). In India, an apparent increase in mucormycosis cases (which was referred to by the misnomer “black fungus”) was attributed to COVID-19 (*3*).

For this investigation, a mucormycosis case was defined as laboratory identification of Mucorales by direct microscopy, culture, or histopathology in a patient with a clinical diagnosis of mucormycosis.^§^ Cases were considered COVID-19–associated if the patient received a positive test result for SARS-CoV-2 (the virus that causes COVID-19) or a COVID-19 diagnosis^¶^ during the period 60 days before to 14 days after mucormycosis diagnosis. Investigators traveled to the four hospitals (three public, and one private) during August 30–September 10, 2021, to ascertain mucormycosis cases and abstract medical record data using a standardized Epi Info (version 7.2.3.1; CDC) case report form. This activity was reviewed by CDC and was conducted consistent with applicable federal law and CDC policy.**

Seventeen persons received a diagnosis of mucormycosis during May 5–September 6, 2021; these included 11 persons with COVID-19–associated cases (Figure). Mucormycosis was confirmed by direct microscopy (16 cases), fungal culture (13 cases), or histopathology (three cases). The demographic features, underlying conditions, and mucormycosis clinical signs and symptoms were similar between patients with and without COVID-19. Most patients were male (nine); the median age was 54 years (IQR = 32–68 years). Diabetes was the most common underlying condition (12 patients), and two patients had hematologic malignancies; no other underlying immunosuppressive medical conditions were noted. During hospitalization, none of the patients with diabetes experienced diabetic ketoacidosis. The most frequent mucormycosis clinical signs and symptoms were rhino-orbital (12 patients) and cutaneous (four patients). The median interval between hospital admission and first positive test result for mucormycosis was 7 days (range = -8 to 21 days). Among the 11 patients with COVID-19–associated mucormycosis cases, nine were unvaccinated against COVID-19; the median interval between COVID-19 diagnosis and the first positive test result for mucormycosis was 11 days (range = -12 to 58 days). Seven COVID-19 patients received supplemental oxygen therapy, nine received corticosteroids, and four received tocilizumab.

**FIGURE:**
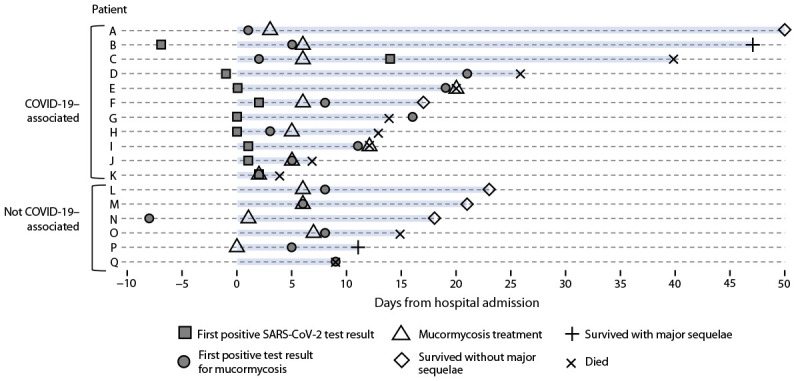
Time line of diagnosis, treatment, and outcomes for patients hospitalized with mucormycosis (N = 17) — Honduras, May–September 2021* * Additional patient information: patient A’s COVID-19 diagnosis date (not included) occurred 58 days before the date of the first positive mucormycosis test result; patients B, F, and M remained hospitalized on the date of data abstraction; and patient K’s date of first positive mucormycosis test result was unavailable.

Ten of the 17 patients died during hospitalization, including eight of the 11 with COVID-19–associated mucormycosis; three patients remained hospitalized at the time of medical chart abstraction. Two of the seven surviving patients experienced major sequelae from mucormycosis, including facial disfiguration and limb loss.

The findings in this report are subject to at least two limitations. First, the actual extent of COVID-19–associated mucormycosis in Honduras is likely underrepresented because case investigations involved only four hospitals in the country. Second, because mucormycosis reporting is not required in Honduras, it is difficult to determine whether the cases described in this report represent an increase over the country’s baseline mucormycosis incidence, which is unknown. The primary laboratory for mycology in Honduras (population approximately 9,900,000)^††^ usually identifies approximately two mucormycosis cases annually (S. Montoya, Hospital Escuela, personal communication, October 2021). By comparison, the 17 mucormycosis cases described in this report occurred during approximately 4 months (May 5–September 6, 2021), coinciding with Honduras’s mid-year COVID-19 surge.^§§^^,^^¶¶^ This apparent increase in laboratory-identified mucormycosis cases might be related to the COVID-19 surge because of COVID-19–induced immune dysregulation or associated medical treatments (*2*). Alternatively, it might reflect the use of an active case-finding strategy during the investigation period. Increased case detection might also be related to higher clinician awareness and testing for mucormycosis, prompted by educational webinars held by SHH, SE-COMISCA, PAHO, and CDC after the initial detection of COVID-19–associated mucormycosis cases in Honduras.

Given the severe outcomes associated with mucormycosis, clinicians should remain vigilant for this disease during the COVID-19 pandemic, including in immunocompetent patients. Early mucormycosis diagnosis is possible, even in resource-limited settings (*4*). Mucormycosis treatment guidelines recommend prompt antifungal therapy*** and surgical intervention to reduce mortality (*4*). Prevention of COVID-19 through vaccination, maintenance of glycemic control in patients with diabetes, and judicious use of steroids^†††^^,^^§§§^ for COVID-19 treatment might help decrease the risk for mucormycosis associated with COVID-19 (*2*). Because of these reported cases, SHH and partners are conducting clinician outreach and education to improve prevention, diagnosis, and treatment of mucormycosis.

## Footnotes

* These authors contributed equally to this report.

§ Signs and symptoms of mucormycosis vary by the affected body site. Rhino-orbital-cerebral mucormycosis signs and symptoms frequently include unilateral facial swelling, headache, sinus congestion, and necrotic lesions of the nasal bridge or palate. Cutaneous mucormycosis signs and symptoms frequently include blisters or ulcers that become necrotic, and pain, erythema, or swelling around a wound. Pulmonary mucormycosis signs and symptoms frequently include cough, chest pain, and shortness of breath. https://www.cdc.gov/fungal/diseases/mucormycosis/symptoms.html

¶ A COVID-19 case was defined as receipt of a positive SARS-CoV-2 reverse transcription–polymerase (RT-PCR) chain reaction or antigen test result, or a clinical diagnosis of COVID-19 in a patient who received a positive serologic test result (RT-PCR or antigen testing was not available in some areas).

** 45 C.F.R. part 46.102(l)(2), 21 C.F.R. part 56; 42 U.S.C. Sect. 241(d); 5 U.S.C. Sect. 552a; 44 U.S.C. Sect. 3501 et seq.

††
https://population.un.org/wpp/Download/Standard/Population/

§§ https://covid19.who.int/region/amro/country/hn (Accessed December 9, 2021).

¶¶ http://covid19honduras.org/(Accessed December 9, 2021).

*** Antifungal drugs that are effective against mucormycosis include amphotericin B, posaconazole, and isavuconazole. Other antifungal drugs, including fluconazole, voriconazole, and echinocandins, are not effective for treating mucormycosis.

†††
https://www.who.int/publications/i/item/WHO-2019-nCoV-Corticosteroids-2020.1

§§§
https://www.covid19treatmentguidelines.nih.gov/management/clinical-management/hospitalized-adults--therapeutic-management/
